# Immunohistochemical Evidence for Glutamatergic Regulation of Nesfatin-1 Neurons in the Rat Hypothalamus

**DOI:** 10.3390/brainsci10090630

**Published:** 2020-09-11

**Authors:** Duygu Gok Yurtseven, Sema Serter Kocoglu, Zehra Minbay, Ozhan Eyigor

**Affiliations:** 1 Department of Histology and Embryology, Bursa Uludag University Institute of Health Science, Bursa 16240, Turkey; dgok@uludag.edu.tr; 2 Department of Histology and Embryology, Balikesir University School of Medicine, Balikesir 10145, Turkey; serter_bio@hotmail.com; 3 Department of Histology and Embryology, Bursa Uludag University School of Medicine, Bursa 16240, Turkey; zminbay@uludag.edu.tr

**Keywords:** glutamate, nesfatin-1, c-Fos, hypothalamus, rat

## Abstract

Nesfatin-1, identified as an anorexigenic peptide, regulates the energy metabolism by suppressing food intake. The majority of nesfatin-1-synthesizing neurons are concentrated in various hypothalamic nuclei, especially in the supraoptic (SON), arcuate (ARC) and paraventricular nuclei (PVN). We tested the hypothesis that the glutamatergic system regulates nesfatin-1 neurons through glutamate receptors. Therefore, the first aim of the proposed studies was to examine effects of different glutamate agonists in the activation of nesfatin-1 neurons using c-Fos double immunohistochemical labeling. Experimental groups were formed containing male and female rats which received intraperitoneal injections of glutamate agonists kainic acid, α-amino-3-hydroxy-5-methyl-4-isoxazolepropionic acid (AMPA) and N-methyl-D-aspartate (NMDA) while the control rats received vehicle. The significant increase in the number of c-Fos-expressing nesfatin-1 neurons after agonist injections were observed both in female and male subjects and some of these effects were found to be sexually dimorphic. In addition, treatment with specific glutamate antagonists 6-cyano-7-nitroquinoxaline-2,3-dione (CNQX) or dizocilpine (MK-801) before each of the three agonist injections caused a statistically significant reduction in the number of activated nesfatin-1 neurons in the hypothalamic nuclei including supraoptic, paraventricular and arcuate nuclei. The second aim of the study was to determine the expression of glutamate receptor subunit proteins in the nesfatin-1 neurons by using a double immunofluorescence technique. The results showed that the glutamate receptor subunits, which may form homomeric or heteromeric functional receptor channels, were expressed in the nesfatin-1 neurons. In conclusion, the results of this study suggest that nesfatin-1 neurons respond to glutamatergic signals in the form of neuronal activation and that the glutamate receptors that are synthesized by nesfatin-1 neurons may participate in the glutamatergic regulation of these neurons.

## 1. Introduction

Hypothalamus is the part of the central nervous system that plays a key role in the hemostasis of the basic functions of the organism. The complex connections and regulatory functions of the peptidergic systems in the hypothalamus have been the subject of intensive research in recent years. Nesfatin-1 was recently identified as an anorexigenic neuropeptide acting in the hypothalamus to control food intake and feeding behavior [1]. Nesfatin-1 is an 82-amino-acid peptide derived from the precursor protein NEFA/nucleobindin 2 (NUCB2) [2,3]. Peripheric or central administration of nesfatin-1 in rats inhibits food intake and causes body weight loss in a dose-dependent manner [1]. It was reported that peripherally injected nesfatin-1 can cross the blood-brain barrier [4,5] and reach the central nervous system. Immunohistochemical studies revealed that the neurons expressing the nesfatin-1 peptide are located in the hypothalamic nuclei, including the supraoptic nucleus (SON), paraventricular nucleus (PVN), arcuate nucleus (ARC), dorsomedial hypothalamic nucleus (DMH), ventromedial hypothalamic nucleus (VMH) and lateral hypothalamic area (LH) [6,7,8,9]. The regulation of food intake through the nesfatin-1 neuronal system is under the control of both peripheral and central signals [1,10,11].

Glutamate is the major excitatory amino acid neurotransmitter that plays an important role in the regulation of neuroendocrine systems in the hypothalamus [12,13]. Glutamate receptors are classified into two groups as ionotropic or metabotropic glutamate receptors. Ionotropic glutamate receptors form ion-specific receptor channels [14] and mediate both excitatory and inhibitory synaptic communication [15]. Subunits of the ionotropic glutamate receptors are extensively expressed by hypothalamic neurons [16]. These receptors are organized under three subfamilies according to their binding affinities of glutamate agonists. N-methyl-D-aspartate (NMDA) receptors are consist of GluN1, GluN2A-D, GluN3A-B subunits, while α-amino-3-hydroxy-5-methyl-4-isoxazolepropionic acid (AMPA) receptors include GluA1-4 subunits and 2-carboxy-3-carboxymethyl-4-isopropenylpyrrolidine (kainate) receptors are made of GluK1-5 subunits [17,18].

The transience expression of c-Fos, the protein product of the c-Fos gene, is used as an indicator to distinguish the activated neurons from non-activated ones in the central nervous system [19,20]. When an acute stimulus is experimentally applied, c-Fos immunoreactivity can be determined in the particular neurons approximately after 60–90 min, and the neurons with c-Fos immunoreactivity in their nuclei are considered as activated neurons [21,22].

There is limited knowledge about the neurotransmitter systems in the control of nesfatin-1 neurons. A recent report showed that the central cholinergic system modulates the effects of nesfatin-1 neurons [23]. Due to the lack of data in the literature demonstrating glutamatergic influences on nesfatin-1 neurons, we aimed to explore the effect of glutamate agonists or antagonists on the activation of these neurons using c-Fos immunohistochemistry. In addition, we analyzed the expression of glutamate receptor subunits in nesfatin-1 neurons using double immunofluorescence technique. The GluN1 and GluN2A subunits of NMDA receptors were included in the experiments, as well as the GluA1, GluA2, GluA3, and GluA4 subunits of AMPA receptors and the GluK1, GluK2, GluK3 and GluK5 subunits of kainate receptors. Three hypothalamic nuclei (SON, PVN and ARC), which contain most of the nesfatin-1 neurons, were analyzed.

## 2. Materials and Methods

### 2.1. Animals

All animal experiments were carried out in accordance with the National Institute of Health Guide for the Care and Use of Laboratory Animals and approved by the Experimental Ethical Committee of Bursa Uludag University (Approval Number: 2014-10/02). Sixty-day-old female and male Sprague–Dawley rats (250–300 g) (*n* = 90, female and male animals), were obtained from the Bursa Uludag University Laboratory Animal Breeding, Usage and Research Center at which the animals were kept in a light- and temperature-controlled facility (a 12:12 h light–dark cycle with lights on at 7:00 am at 21 °C) with food and water freely available (ad libitum). The rats were fasted overnight before the day of the experiment in order to minimize the activation of nesfatin-1 neurons.

The estrous cycles of the female rats were daily controlled via the evaluation of vaginal smears (three cycles), and all experiments were performed on the morning of diestrus-I (metestrus) with the lowest gonadal hormone levels [24]. Thus, the possible interference of different hormone profiles of female animals to affect the results obtained from the experiments was minimized.

### 2.2. Experimental Groups and Injections

Three main experimental groups were designed to demonstrate the effect of different glutamate receptor agonists (kainic acid, AMPA and NMDA) on the activation of nesfatin-1 neurons. There were 30 animals in each main group—designated as kainic acid, AMPA and NMDA groups. Each main group was divided into three subgroups as agonist, control and antagonist groups (*n* = 10 per subgroup, 5 male and 5 female). Kainic acid agonist group received an intraperitoneal (i.p.) injection of AMPA/kainate receptor agonist kainic acid (2.5 mg/kg in 300 μL distilled water, DW). The kainic acid control group received saline injections (300 μL saline, i.p.). The rats in the kainic acid antagonist group were injected with CNQX, a kainate/AMPA receptor antagonist (2 mg/kg in 300 μL DW, i.p.), 15 min before the agonist injection as described above. The rats in the AMPA agonist group were injected with AMPA (5 mg/kg in 750 μL DW, i.p.). The AMPA control group got intraperitoneal saline injections (750 μL). In the AMPA antagonist group, the rats received CNQX injections (2 mg/kg in 300 μL DW, i.p.) 15 min prior to the AMPA injections. In the NMDA agonist group, NMDA was intraperitoneally given (100 mg/kg in 2 mL DW), and the NMDA control group was injected with 2 mL saline (i.p.). MK-801 (1 mg/kg in 300 μL DW, i.p.) was administered to the rats in the NMDA antagonist group 15 min before the NMDA injection.

All injections were performed between 9:00 and 13:00. Ninety minutes after the last injection, both injected and intact animals were deeply anesthetized with ether inhalation and fixed by transcardial perfusion with 4% paraformaldehyde (PFA) in 0.13 M Sorenson’s phosphate buffer, pH 7.4 (350 mL/animal). All brains were removed and post-fixed at +4 °C overnight in the same fixative. Forty-micrometer-thick serial coronal sections throughout the hypothalamus including SON, ARC and PVN were cut with a vibratome and collected in 5 consecutive series of glass vials containing Tris-HCl buffer (0.05 M, pH 7.6). After thoroughly washing in Tris-HCl buffer, the sections were kept in cryoprotectant solution at −20 °C until use.

### 2.3. Immunohistochemistry

For all washing steps, with the exception of blocking and primer antibody incubation, Tris-HCl buffer (0.05 M, pH 7.6) was used. Blocking step was carried out with a buffer including 10% normal horse serum, 0.1% sodium azide, and 0.2% Triton X-100 in Tris-HCl and this buffer was also used for the dilution of primary and secondary antibodies. An orbital shaker with appropriate agitation was used for all incubation and washing steps.

First c-Fos, and then nesfatin-1 protein, was consecutively labeled on the same brain sections by using the double immunohistochemical method. Free-floating sections were used for immunohistochemistry after the removal of the cryoprotectant in Tris-HCl. Then, the blocking step was performed for 2 h in blocking buffer. Sections were then incubated in rabbit anti-c-Fos (1:10,000, Chemicon, Billerica, MA, USA) antibody overnight at room temperature. A two-hour incubation in secondary antibody solution containing biotin-SP-conjugated donkey anti-goat IgG, (1:300, Jackson Immunoresearch Labs, West Grove, PA, USA) followed the primary antibody incubation. The sections were processed with an avidin–biotin complex (ABC Elite Standart Kit, Vector Labs, Burlingame, CA, USA) for 60 min. Nickel-intensified diaminobenzidine (DAB) solution (1 g nickel ammonium sulfate, 12.5 mg DAB, in 50 mL Tris-HCl buffer with 1.3 μL hydrogen peroxide) was used to stain the c-Fos immunoreactivity in the nuclei. Following the c-Fos staining the same section set was processed for nesfatin-1 immunohistochemistry. Washed and blocked sections were incubated in rabbit anti-nesfatin-1 antibody (1:20,000, Phoenix Pharmaceuticals, H-003-22, Burlingame, CA, USA) overnight at room temperature. Nesfatin-1 antibody specificity is shown in previous studies in the literature [6,7,8,9]. After the primary antibody incubation, the same procedure was followed as explained above and the reaction was made visible by substrate–chromogen solution (50 mg DAB, 5 μL hydrogen peroxide in 100 mL Tris-HCl buffer). Double-stained sections were washed, collected onto glass slides, dried and cover-slipped with DPX. Negative control experiments included omission of primary or secondary antibodies, which revealed no specific staining.

### 2.4. Immunofluorescence

Washing and blocking steps were applied as described above. Before the primary antibody incubation, sections were pre-treated with 50 mM trisodium citrate solution (pH 6, +80 °C) for antigen retrieval. The free-floating sections were incubated in the primary antibody mixture including nesfatin-1 antibody (rabbit anti-nesfatin-1, 1:20,000, Phoenix Pharmaceuticals, H-003-22, Burlingame, CA, USA) and the primary antibody against one of the glutamate receptor subunits at +4 °C. Dilutions, incubation times and incubation temperatures, as well as the supplier and catalog number information of the primary antibodies used in this study, are given in Table 1.

Following the primary antibody incubation, sections were exposed to the mixture of secondary antibodies. All secondary antibodies were diluted in blocking buffer and incubation time was 2 h. Alexa Fluor 488 conjugated donkey anti-rabbit IgG (1:400, Thermo Fisher Scientific, Rockford, IL, USA) was used to visualize nesfatin-1 signals. Alexa Fluor 594 conjugated donkey anti-mouse IgG (1:400, Thermo Fisher Scientific, Rockford, IL, USA) was used for all the receptor subunit signals except GluA4 and GluK1/2/3. Alexa Fluor 594 conjugated donkey anti-goat IgG (1:400, Thermo Fisher Scientific, Rockford, IL, USA) was used for GluA4 signal. In order to detect the signal for the GluK1/2/3 antibody (which is in the form of IgM) and recognize all three forms of kainate receptors, sections were first incubated in biotin-conjugated donkey anti-mouse IgM (1:300, Jackson Immunoresearch, West Grove, PA, USA) for 2 h followed by an incubation in Texas red-conjugated streptavidin (1:100, Jackson Immunoresearch, West Grove, PA, USA) for another 2 h. Sections were then washed, collected onto glass slides, dried, and mounted with Prolong Antifade (Molecular Probes, Eugene, OR, USA) before microscopic evaluation.

### 2.5. Cell Counting and Statistical Analysis

For the analyses of the stained sections, as well as for capturing the digital images, a microscope (BX50, Olympus Corporation, Tokyo, Japan) equipped with a reflected light fluorescence attachment (BX-FLA, Olympus Corporation, Tokyo, Japan) and a digital camera (Olympus DP-71 CCD color camera, 1.5 million pixels, Olympus Corporation, Tokyo, Japan) were used. Sections including SON (between bregma −0.48 mm and −1.44 mm), PVN (between bregma −1.32 mm and −1.92 mm) and ARC (between bregma −1.80 mm and −2.76 mm) were analyzed for the presence of immune-positive nesfatin-1 neurons [25].

Cell counting (without random sampling) was manually performed bilaterally (using 40× objective) on five different consecutive sections for each animal taken from the same coordinates according to the brain atlas by Paxinos and Watson [25]. First, all immunopositive nesfatin-1 neurons (neurons with brown cytoplasmic staining) were counted in the respective hypothalamic nucleus. Then the presence of c-Fos reaction (black staining in the nucleus) in these nesfatin-1-labeled neurons was recorded. A neuron was considered double labeled if it possessed both brown cytoplasmic (nesfatin-1 reaction) and black nuclear (c-Fos reaction) labeling. Cell counting was performed by two different observers who were blind to the experiments. Later on, the ratio of the number of c-Fos-positive nesfatin-1 neurons to the number of all neurons with nesfatin-1 immunoreaction was calculated for each animal. For each group, means and standard deviations of the percentages obtained from each animal were determined. While the mean differences between the experimental groups were analyzed using one-way analysis of variance (ANOVA), gender differences were analyzed by two-way analysis of variance, and followed by Tukey post hoc test. Differences between two groups were assessed using Student’s t-test and the *p* < 0.05 was set as the statistical significance value.

For the immunofluorescence applied sections, each nesfatin-1 neuron was analyzed in order to detect the expression of the particular glutamate receptor subunit. For each receptor subunit, sections from 5 different male rats were analyzed and the population of double-labeled nesfatin-1 neurons was noted. Data were collected by two observers blind to the experiment, and semi-quantitative scoring was performed. Thus, if most of the nesfatin-1 neurons in the particular nucleus express the respective subunit, a scoring of “+++” was used, while “++” was used for a moderate number of neurons or “+” was used if a few numbers of neurons were double labeled.

## 3. Results

### 3.1. Nesfatin-1, c-Fos and Glutamate Receptor Immunoreactivity in the Hypothalamic Neurons

**Figure 1:**
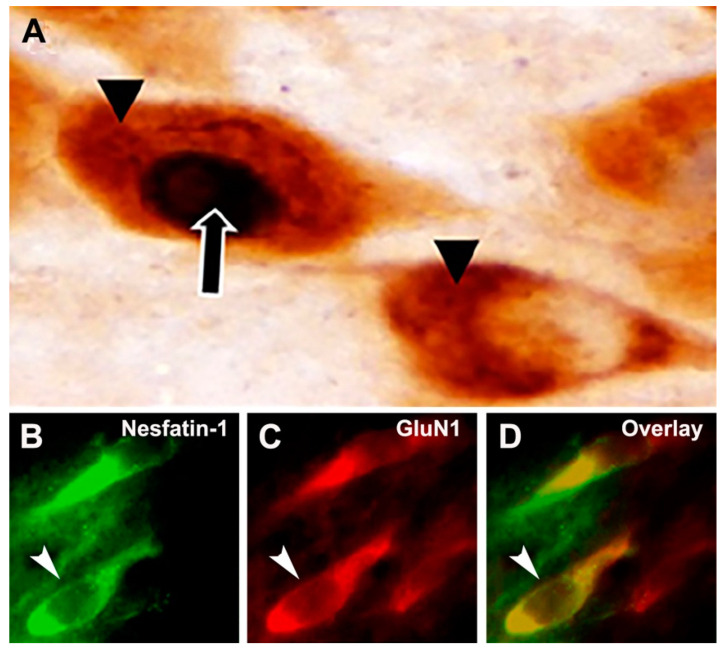
General representations of the immunohistochemical stainings in this study. (**A**) In the technique of double indirect immunoperoxidase labeling, immunoreactive nesfatin-1 neurons (▲) were detected by the presence of brown precipitation in the cytoplasm, whereas the c-Fos immunoreaction (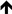) was localized in the nucleus with a black chromogen reaction, so the active nesfatin-1 neurons are distinguished by the presence of c-Fos-positive nuclei. (**B**) Nesfatin-1 neurons are labeled with green fluorochrome. (**C**) Glutamate receptor subunit (GluN1 as the example in this figure) proteins are labeled with red fluorochrome. (**D**) Nesfatin-1 neurons expressing GluN1 subunit protein are monitored in yellow. Arrows point out a double-labeled neuron.

Nesfatin-1 immunoreactivity was detected in the neuronal cytoplasm as brown staining (DAB reaction). Microscopic analyses showed that nesfatin-1-expressing neurons were localized in the supraoptic, arcuate, paraventricular and periventricular nuclei and the lateral area of the hypothalamus. c-Fos immunoreactivity was localized in the nucleus of the neurons with a blue-black color (nickel-enhanced DAB reaction (Figure 1A). Double-labeled hypothalamic neurons that were immune-positive for both proteins were considered as activated neurons.

In double immunofluorescence staining, neurons which express nesfatin-1 protein were detected in green color (Alexa Fluor 488), while the neurons containing glutamate receptor subunit proteins were identified with red color (Alexa Fluor 594 or streptavidin-conjugated Texas Red). When the images digitally superimposed, the double-labeled neurons were seen in yellow color (as an example; Figure 1B–D).

### 3.2. Effects of Glutamatergic Agonists and Antagonists on the Activation of Nesfatin-1 Neurons

#### 3.2.1. Supraoptic Nucleus

**Figure 2:**
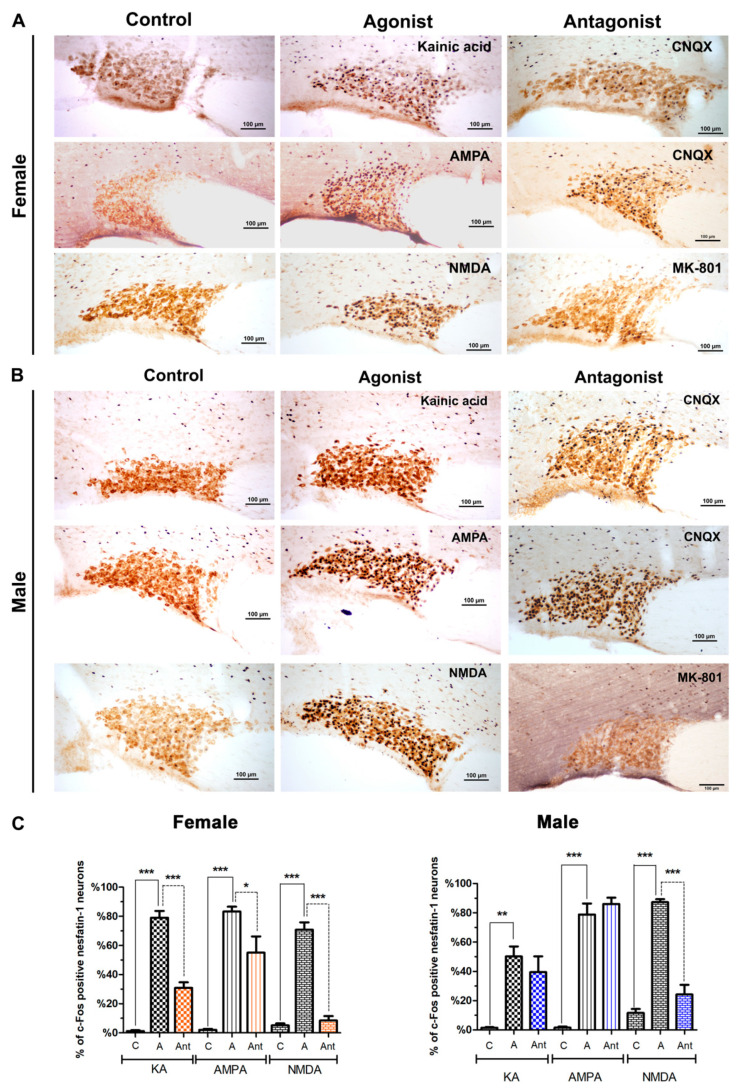
Representative images of the effect of glutamate agonists and antagonists on nesfatin-1 neurons localized in the supraoptic nucleus of female (**A**) and male (**B**) rats. The effect of kainic acid, α-amino-3-hydroxy-5-methyl-4-isoxazolepropionic acid (AMPA), and N-methyl-D-aspartate (NMDA) is shown. Control groups are shown on the left, the agonist injected groups in the middle, and antagonist injected groups on the right. Nesfatin-1 (brown reaction) and c-Fos (black reaction) antibodies were used for this experiment. Figure (**C**) summarizes the percentages of c-Fos-positive nesfatin-1 neurons relative to all nesfatin-1-positive cells in the supraoptic nucleus (SON). Asterisk indicates the statistical significance between control and agonist groups, as well as between agonist and antagonist groups (*n* = 5/group, * *p* < 0.05, ** *p* < 0.01, *** *p* < 0.001, C: Control, A: Agonist, Ant: Antagonist).

We found that all three glutamatergic agonists (kainic acid, AMPA and NMDA) increased the number of activated nesfatin-1 neurons in the supraoptic nucleus when compared to their respective control groups. In female rats, about 79% of the nesfatin-1 neurons were c-Fos-positive following kainic acid injection and about 83% were activated by AMPA. NMDA injection caused neuronal activation in about 70% of the nesfatin-1 neurons. When these data were compared with the mean percentage of activated nesfatin-1 neurons in the control groups (for kainic acid 1%, for AMPA 2%, for NMDA 5%), the increase in the number of activated nesfatin-1 neurons was found to be statistically significant for all three agonists (*p* < 0.001). When the specific antagonist was injected prior to the agonist injection, the neuronal activation was partially blocked. Although the antagonist application did not reduce the number of c-Fos -positive nesfatin-1 neurons to the control levels, a statistically significant decrease was detected for all antagonist experiments (30% by kainic acid + CNQX, 55% by AMPA + CNQX, and 8% by NMDA + MK-801 (Figure 2A).

In males, the ratio of c-Fos-positive nesfatin-1 neurons, which was 1% in control group, increased to 50% following kainic acid injection, and decreased to 39% by the antagonistic effect of CNQX. AMPA application caused a significant increase in neuronal activation since the percentage of double-labeled nesfatin-1 neurons was increased from 1% in the control group to 78%. Administration of CNQX before the AMPA injection revealed no antagonistic effect. The most effective blocking was seen with MK-801, the NMDA receptor antagonist, which decreased the percentage of NMDA-activated nesfatin-1 neurons from 87% to 24%. The increase in the number of c-Fos-expressing nesfatin-1 neurons after NMDA injection was proved to be statistically significant when compared to the control group which had a ratio of 11% activation (Figure 2B). The graphical representation of the data collected from the supraoptic nucleus is provided in Figure 2C.

#### 3.2.2. Paraventricular Nucleus

**Figure 3:**
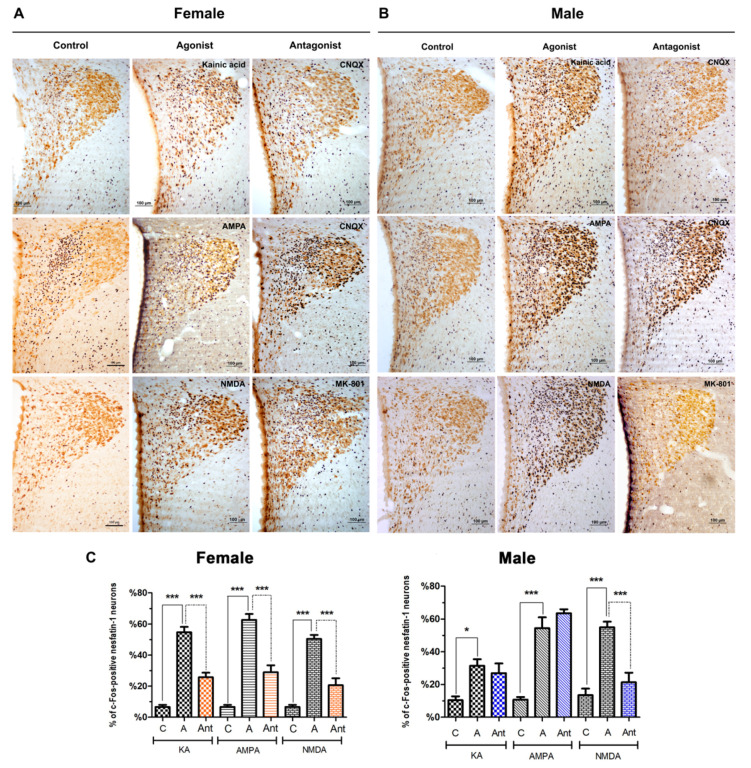
Representative images of the effect of glutamate agonists and antagonists on nesfatin-1 neurons localized in the paraventricular nucleus of female (**A**) and male (**B**) rats. The effect of kainic acid, AMPA, and NMDA is shown. Control groups are shown on the left, the agonist injected groups in the middle, and antagonist injected groups on the right. Nesfatin-1 (brown reaction) and c-Fos (black reaction) antibodies were used for this experiment. Figure (**C**) summarizes the percentages of c-Fos-positive nesfatin-1 neurons relative to all nesfatin-1-positive cells in the paraventricular nuclei (PVN). Asterisk indicates the statistical significance between control and agonist groups, as well as between agonist and antagonist groups (*n* = 5/group, * *p* < 0.05, *** *p* < 0.001, C: Control, A: Agonist, Ant: Antagonist).

In the paraventricular nucleus of the female rats, about 54% of nesfatin-1 neurons expressed c-Fos following kainic acid injection, whereas this ratio was 62% after AMPA and 50% after NMDA. When compared with the respective control groups, the increase in the number of active neurons was statistically significant (*p* < 0.001). The injections of specific antagonists before the agonist administration, significantly blocked the agonistic effect and decreased the percentage of c-Fos-positive nesfatin-1 neurons to 25% in kainic acid + CNQX group, 28% in AMPA + CNQX and 20% in NMDA + MK-801 group (Figure 3A).

When compared to the control groups, the proportion of the double-labeled neurons in the male rats showed a statistically significant increase in all three groups injected with the particular agonist. Thus, the number of c-Fos+/nesfatin-1 + cells increased after kainic acid injection (31% versus 10% in control, *p* < 0.05), AMPA injection (54% versus 10% in control, *p* < 0.001) and NMDA injection (54% versus 13%, *p* < 0.001). Administration of CNQX before kainic acid or AMPA injections exhibited no statistical difference in the number of activated neurons, whereas injection of MK-801 significantly decreased the percentage of c-Fos-positive nesfatin-1 neurons to 21% (Figure 3B). The graphical representation of the data collected from the paraventricular nucleus is provided in Figure 3C.

#### 3.2.3. Arcuate Nucleus

**Figure 4:**
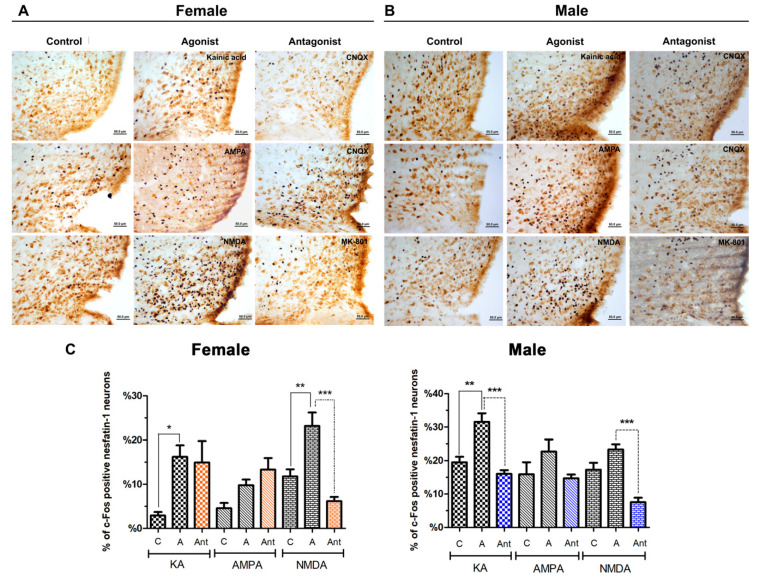
Representative images of the effect of glutamate agonists and antagonists on nesfatin-1 neurons localized in the arcuate nucleus of female (**A**) and male (**B**) rats. The effect of kainic acid, AMPA, and NMDA is shown. Control groups are shown on the left, the agonist injected groups in the middle, and antagonist injected groups on the right. Nesfatin-1 (brown reaction) and c-Fos (black reaction) antibodies were used for this experiment. Figure (**C**) summarizes the percentages of c-Fos-positive nesfatin-1 neurons relative to all nesfatin-1-positive cells in the arcuate (ARC). Asterisk indicates the statistical significance between control and agonist groups as well as between agonist and antagonist groups (* *p* < 0.05, ** *p* < 0.01, *** *p* < 0.001, C: Control, A: Agonist, Ant: Antagonist).

In the arcuate nucleus of female rats, both kainic acid (16% versus 2%, *p* < 0.05) and NMDA (23%, versus 11%, *p* < 0.01) significantly increased the number of activated neurons compared with the control group, but in the AMPA group the increase was not significant (9% versus 4%, *p* = 0.22). When the number of activated nesfatin-1 neurons was assessed, injection of CNQX showed no significant blocking of the effects of kainic acid or AMPA. Significant difference was only detected between the NMDA group and NMDA + MK-801 group (23%, 6%, respectively, *p* < 0.001) (Figure 4A).

In the male rats, the percentages of c-Fos-positive nesfatin-1 neurons were about 31% following kainic acid treatment, 22% after AMPA and 23% for NMDA. Respective to the agonist groups, active neuronal rate in control groups were 19%, 15% and 17%. Statistical comparison revealed significant increase in kainic acid and NMDA injected animals. Although more nesfatin-1 neurons were activated after AMPA administration, this increase was not statistically significant. The injections of specific antagonists caused a statistically significant decrease in the activation of nesfatin-1 neurons in kainic acid + CNQX (16%, *p* < 0.001), and NMDA + MK-801 groups (7%, *p* < 0.001) (Figure 4B). The graphical representation of the data collected from the arcuate nucleus is provided in Figure 4C.

**Figure 5:**
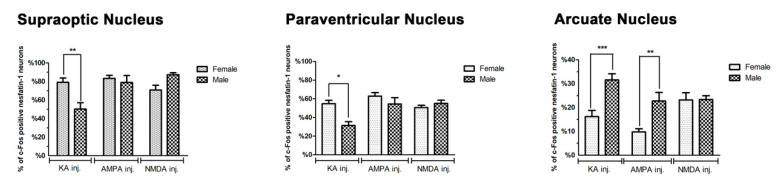
The figure summarizes the gender-related differences in the percentages of c-Fos-positive nesfatin-1 neurons relative to all nesfatin-1-positive cells in all the hypothalamic nuclei analyzed. Asterisk indicates the statistical significance between control and agonist groups, as well as between agonist and antagonist groups (* *p* < 0.05, ** *p* < 0.01, *** *p* < 0.001).

When the gender-related differences on the effects of glutamate agonists were analyzed, results showed that kainic acid activated significantly more nesfatin-1 neurons in the SON of female rats when compared to male rats. AMPA or NMDA effects showed minimal differences without statistical significance in both genders (*p* < 0.01, Figure 5). In the PVN, it was determined that only the kainic acid effect on neuronal activation was significantly higher in female rats than male rats (*p* < 0.05). No statistically significant differences were observed between female and male animals for AMPA and NMDA groups (Figure 5). In the arcuate nucleus, the proportion of activated nesfatin-1 neurons in male animals was significantly higher than that of females following kainic acid or AMPA injections (*p* < 0.001 and *p* < 0.01), respectively. The stimulatory effect of NMDA was found to be similar in both gender (Figure 5).

In addition, when we analyzed the data in terms of the potency of the glutamate agonists in the activation of nesfatin-1 neurons, significant differences were observed in the SON and PVN of male rats and the ARC of the female rats. In males, AMPA and NMDA caused activation in about two-fold more nesfatin-1 neurons in the SON and PVN. In the ARC of the female rats, NMDA was found to be more effective than kainic acid or AMPA.

### 3.3. Expression of Glutamate Receptor Subunits in Nesfatin-1 Neurons

**Figure 6:**
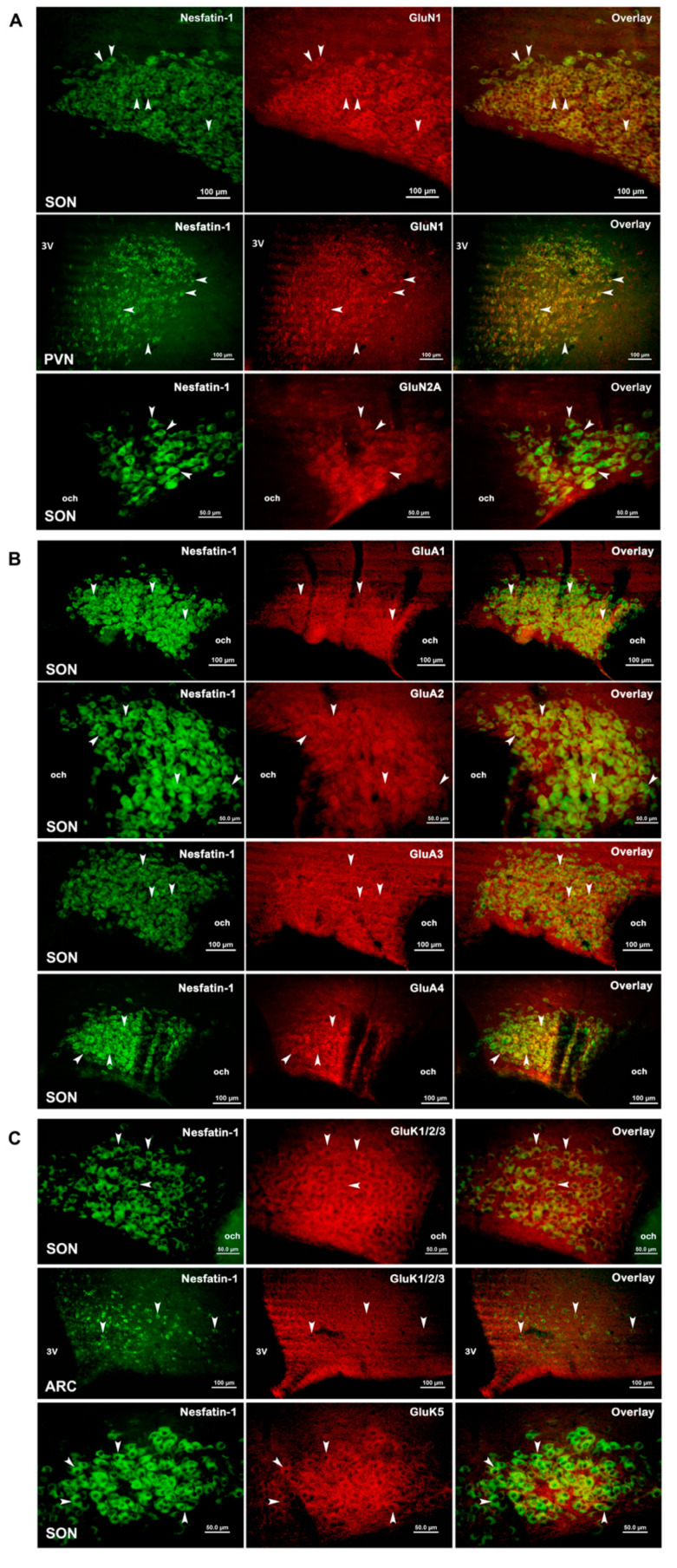
(**A**) Immunofluorescence images of GluN1 and GluN2A subunit protein expression of NMDA receptors in nesfatin-1 neurons which are localized in SON and PVN. (**B**) Immunofluorescence images of GluA1, GluA2, GluA3 and GluA4 subunit protein expression of the AMPA receptors in nesfatin-1 neurons, which are localized in SON. (**C**) Immunofluorescence images of GluK1/2/3 and GluK5 receptor protein expression in nesfatin-1 neurons which are localized in SON and ARC. While nesfatin neurons were marked with Alexa 488 seen in green, glutamate receptor subunits were marked with the Alexa 594 and are seen in red color. Neurons expressing both nesfatin-1 and glutamate receptor protein are seen in yellow in the overlay panel. Arrows show the examples of double-labeled nesfatin-1 neurons with the particular GluR; 3V: third ventricle; och: optic chiasm.

Microscopic analyses showed that all glutamate receptors are expressed in most of the nesfatin-1 neurons localized in the supraoptic nucleus. It was determined that the GluN1 subunit of the NMDA receptor subfamily was present in most of the nesfatin-1 neurons in the SON. GluN1 protein expression was present in moderate number of nesfatin-1 neurons in PVN or ARC and a few in the lateral hypothalamus. GluN2A subunit was also present in high number of nesfatin-1 neurons in the SON, while a few neurons in the PVN and ARC expressed GluN2A (Figure 6A).

In the supraoptic nucleus, GluA1, GluA2 and GluA3 subunits of the AMPA receptors were detected in most of the nesfatin-1 neurons, while GluA4 subunit was observed in a moderate number of nesfatin-1 neurons. Moderate number of nesfatin-1 neurons were GluA2- and GluA4-positive in the PVN. Few nesfatin-1 neurons in the paraventricular nucleus exhibited immunohistochemical signals for GluA1 or GluA3. AMPA receptor subunits were seen in few numbers of nesfatin-1 neurons in the arcuate nucleus (Figure 6B).

In the SON, the staining with the antibody recognizing all of the low-affinity kainate receptor subunits (GluK1, GluK2 and GluK3) indicated that large numbers of nesfatin-1 neurons were labeled with this antibody. In contrast, few neurons were double labeled in the PVN or ARC. Double immunofluorescence studies have shown that most of the nesfatin-1 neurons are labeled with the antibody recognizing kainate-preferring GluK5 subunit protein in the SON, PVN and ARC (Figure 6C). The results of the semi-quantitative analyses are summarized in Table 2.

## 4. Discussion

### 4.1. Effects of Glutamatergic Agonists and Antagonists on the Activation of Nesfatin-1 Neurons

The distribution pattern of the nesfatin-1 neurons in the central nervous system was first described by Oh-I et al. [1]. Further studies confirmed the preferential localization of nesfatin-1 neurons in the hypothalamic nuclei [7,8,9]. In accordance to the previous studies in the literature, we detected nesfatin-1-immunoreactive neurons in the supraoptic nucleus, paraventricular nucleus, arcuate nucleus, periventricular nucleus and lateral hypothalamic area. The present study was focused on the nesfatin-1 neurons in the SON, PVN and ARC only. Although this seems to limit the results of the study, it is known that the vast majority of the nesfatin-1 neurons in the central nervous system are found in these three hypothalamic nuclei.

Glutamatergic system plays an important role in the regulation of many neuroendocrine and peptidergic systems in the hypothalamus [13]. The ionotropic glutamate agonists selectively bind to different types of glutamate receptors and transmit their stimulation to neurons and provide neuronal activation [26]. It has been reported that glutamate agonists, including kainic acid [27,28,29], AMPA [30,31], and NMDA [31,32,33,34], can reach the central nervous system by passing the blood-brain barrier. The present study showed that these agonists can reach the nesfatin-1 neurons in the hypothalamus. In the literature, there is a lack of information on the effects of major neurotransmitter systems in the regulation of nesfatin-1 neurons. The present study revealed evidence that the glutamate agonists may bind to their selective receptors expressed in the nesfatin-1 neurons and trigger neuronal activation. In addition to this possible direct effect, agonist signals may indirectly reach nesfatin-1 neurons through an unidentified pathway which might include other neurotransmitter and/or neuromodulator systems. Our experimental approach limits us to distinguish between these possibilities.

In the supraoptic nucleus, the percentage of c-Fos-positive nesfatin-1 neurons was determined in kainic acid-, AMPA-, and NMDA-injected female rats to be 79%, 83%, and 70%, respectively. These 45 to 75-fold increases were statistically significant when compared to controls. Thus, all three agonists provide activation by increasing basal c-Fos expression in nesfatin-1 neurons located in the SON. Since transient expression of c-Fos protein can be used as a neuronal activation marker [21], it is possible to say that a genetic activity following this activation, which is induced by glutamate, may lead to the synthesis and/or secretion of nesfatin-1 peptide. This possibility was not evaluated in the present study. Previous studies performed in our laboratory similarly showed that glutamate agonists activate oxytocin, vasopressin, atrial natriuretic peptide, orexin or neuronostatin neurons and that these neurons express glutamate receptors [27,35,36,37]. In male rats, the percentage of active nesfatin-1 neurons interestingly varied according to the effect of the agonists. NMDA seemed to be the most potent agonist which increased the basal c-Fos expression levels in the nesfatin-1 neurons in males, while AMPA has the most potent effect in females. The results also showed that kainic acid is more effective in females than males, suggesting a possible sexually dimorphic regulatory mechanism for glutamate in controlling nesfatin-1 neurons. Different sex-related interactions are seen especially in neuroendocrine systems [38].

In the PVN, all three agonists induced an increase in the percentage of c-Fos+/nesfatin-1+ neurons by 9 to 10-fold in female rats, showing the PVN nesfatin-1 neurons also respond the glutamatergic signals. The percentage of double-labeled immune-reactive neurons in the male subjects showed a significant increase in all three groups injected with the agonist when compared to the control groups. Similar to SON, kainic acid activates a significantly higher number of nesfatin-1 neurons in female rats in the PVN when compared to male rats.

In the arcuate nucleus, the basal c-Fos expression in the control group was higher in nesfatin-1 neurons compared to SON or PVN. This suggests that the nesfatin-1 neurons in the ARC may differ in function than the rest of the population. Despite the high basal values, the injection of glutamate agonists also activated a significantly greater number of nesfatin-1 neurons in the ARC. Additionally, we found that while the most effective agonist was NMDA in females, kainic acid was most powerful in males. These results suggest that glutamate demonstrate different regulation on nesfatin-1 neurons in the arcuate nucleus, compared to SON and PVN nesfatin-1 neurons.

The results showed similarities between SON and PVN, but differences in the ARC. Although our experimental approach was not designed to understand the functional importance of this dissimilarity in the ARC, we can speculate that the nesfatin-1 neurons in the ARC are functionally different than the neurons in the SON or PVN. Some of these functional differences related to the distinct systems of the organism are documented in the literature [10,39].

Our results show that agonist effects are significantly suppressed by appropriate antagonists. Thus, MK-801 has the strongest effect in the supraoptic nucleus of female rats, because it decreased the number of active nesfatin-1 neurons to the level of the control group. CNQX, both kainic acid and AMPA antagonist, was found to be effective in kainate receptor antagonism in contrast to AMPA receptors. The partial blockage of the agonist effects suggests that the glutamatergic signals directly and/or indirectly reach nesfatin-1 neurons through functional glutamate receptors.

These data suggest that glutamate is a potent regulator of the nesfatin-1 neurons in the hypothalamus. This regulation may be towards the different functions of nesfatin-1 peptide including, but not limited to, the suppression of food intake, cardiovascular regulation and energy expenditure [10]. A recent report suggested that the central cholinergic system may participate in the modulation of the effects of nesfatin-1 on the cardiovascular activity [23]. In addition, recent unpublished data from our laboratory showed that the glutamatergic system plays an important role in the modulation of stress responses of the nesfatin-1 neurons. Thus, glutamatergic antagonists decreased the number of activated nesfatin-1 neurons during stress. In light of this knowledge, we can speculate that glutamatergic signals may possess a regulatory mechanism on stress-induced anorexia and/or in stress-induced activation of the hypothalamic-pituitary-adrenal axis. However, further studies are needed to clarify this mechanism. To our knowledge, this present report is the first in the literature showing that glutamate, one of the classical neurotransmitters, exerts its effects on the nesfatin-1 neurons.

It is important to implicate that the immunohistochemistry is limited to the detectable levels of the proteins that are analyzed in this study. Our results only include the nesfatin-1 neurons that can be identified by immunohistochemistry, and the numbers provided in this study only represent these neurons.

### 4.2. Expressions of Glutamate Receptor Subunit Proteins in Nesfatin-1 Neurons

It is well established that glutamate is the primary amino acid neurotransmitter in the glutamatergic neurotransmission and binds glutamate receptors with different subunit compositions [12,13,19,40]. Data obtained using in situ hybridization technique, as well as immunohistochemistry, have shown that subunit proteins belonging to all three subfamilies of glutamate receptors were expressed in the hypothalamus. It is reported that GluN1 from the NMDA receptor family, GluA1 from the AMPA receptor family and GluK5 subunit from the kainate receptor family are the most common subunits in the hypothalamus [16,41,42,43,44]. In accordance, the present study revealed that these subunits are expressed by most of the nesfatin-1 neurons in the SON, PVN and ARC. Immunohistochemical studies have shown that GluN1 [45], GluN2A and GluN2B receptor proteins [44,46] are expressed in high density in the magnocellular neurons localized in the SON and PVN, but no data showing the expression of GluN3A-B subunits in both nuclei. As a result of a study investigating the presence of kainate receptor subunits in the supraoptic nucleus at the mRNA level, it was shown that GluK5 is expressed at very high density and GluK1 at low density [16]. Expression of GluK4 was absent in the hypothalamus [16]. In regard to these reports, GluN3 and GluK1 subunits were not included in our study.

The results of our study showed that both NMDA and non-NMDA glutamate receptor proteins were synthesized by nesfatin-1 neurons localized in the hypothalamus. In the SON, most nesfatin-1 neurons express all the subunits included in this study. This suggests that the supraoptic nuclei might be the primary site of action for the glutamatergic regulation of nesfatin-1 neurons. While the expression of the GluN1 subunit of the NMDA receptors, GluA1 and GluA2 subunits of the AMPA receptors and the GluK5 subunit of the kainate receptors were determined in most nesfatin-1 neurons by double immunofluorescence, other subunits were found to be expressed in a smaller number of nesfatin-1 neurons in the hypothalamus. Glutamate receptors become functional by forming homomeric complexes with the same subunits or heteromeric receptor complexes with different subunits [47,48,49,50,51]. To this knowledge, our study suggested that nesfatin-1 neurons could possess functional glutamate receptors in SON, PVN and ARC, since they express most of the necessary subunits in order to form functional receptor channels. Thus, GluK5, which cannot form channels alone, may combine with one of the GluK1, GluK2 or GluK3 subunits to form functional heteromeric kainate-selective receptors in nesfatin-1 neurons [47,48]. Low affinity subunits (GluK1, GluK2 and GluK3) may form homomeric kainate receptors [48]. Similarly, GluA1 or GluA2 subunits, which we have shown to be expressed in nesfatin-1 neurons, can form homomeric and/or heteromeric functional AMPA-selective glutamate receptors [49]. In addition, the expression of GluA3 and GluA4 subunits in the nesfatin-1 neurons is important since these subunits can only form heteromeric glutamate receptors with the presence of GluA1 and/or GluA2 subunits.

Another important finding is that GluN1 protein, which is the indispensable subunit of NMDA receptors [49], is also expressed in nesfatin-1 neurons. Since nesfatin-1 neurons can synthesize the GluN2A subunit, together with the GluN1 subunit, they can form functional NMDA-selective glutamate receptors in the presence of these two subunits.

Although our analyses on the expression of the glutamate receptors in the nesfatin-1 neurons produced a semi-quantitative data, it clearly shows that these neurons possess the genetic mechanisms to synthesize ionotropic glutamate receptor subunits. Our laboratory is limited to the classical fluorescence microscopy techniques and we are aware of the possibility of obtaining fully quantitative results if other analysis systems such as confocal microscopy could have been used.

In conclusion, the results of this study suggest that nesfatin-1 neurons synthesize glutamate receptors and are activated by glutamate agonists. These neurons express glutamate receptor subunits which are necessary to form homomeric and/or heteromeric functional receptor channels. This implies that endogenous glutamate can bind to its receptors on nesfatin-1 neurons and exert its regulatory effects on these neurons, and there may be different sex-dependent interactions between male and female rats in this effect. In turn, this excitatory signal may trigger the actions of nesfatin-1 on the suppression of nutrient uptake. Further physiological and/or pharmacological studies would be needed in order to demonstrate the possible usage of glutamatergic neurotransmission as a target mechanism for clinical studies of the food intake disorders.

## Figures and Tables

**Table 1:** Properties of the primary antibodies.

Antibodies	Dilution	Incubation Time	Temperature	Supplier	Catalog Number
Rabbit anti-c-Fos	1:10,000	24 h	RT	Oncogene	PC-38
Rabbit anti-Nesfatin-1	1:20,000	24 h	RT	Phoenix Pharmaceuticals	H-003-22
Mouse anti-GluN1 (IgG)	1:300	48 h	+4 °C	BD Pharmingen	556,308
Mouse anti-GluN2A (IgG)	1:1,000	48 h	+4 °C	Millipore	MAB5216
Mouse anti-GluA1 (IgG)	1:500	48 h	+4 °C	Acris	AM60040PU-N
Mouse anti-GluA2 (IgG)	1:1,000	48 h	+4 °C	Millipore	MAB397
Mouse anti-GluA3 (IgG)	1:1,000	48 h	+4 °C	Millipore	MAB5416
Goat anti-GluA4 (IgG)	1:500	48 h	+4 °C	LifeSpan BioSciences	LS-B3606
Mouse anti-GluK1/2/3 (IgM)	1:900	48 h	+4 °C	Chemicon	MAB379
Goat anti-GluK5 (IgG)	1:2,000	72 h	+4 °C	Santa Cruz	sc-8915

Dilutions, incubation times and temperatures were optimized by preliminary staining. RT: room temperature.

**Table 2:** Semi-quantitative analyses of double-labeled nesfatin-1 neurons with glutamate receptor subunits in the hypothalamus.

	Hypothalamic Nuclei		
Subunits	Supraoptic Nucleus	Paraventricular Nucleus	Arcuate Nucleus
GluN1	+++	++	++
GluN2A	+++	+	+
GluA1	+++	+	++
GluA2	+++	++	+
GluA3	+++	+	+
GluA4	++	++	+
GluK1/2/3	+++	+	+
GluK5	+++	++	++

+: low number of neurons, ++: moderate number of neurons, +++: high number of neurons.

## Abbreviations

ABC	Avidin-biotin complex
AMPA	α-amino-3-hydroxy-5-methyl-4-isoxazolepropionic acid
ARC	Arcuate nucleus
DAB	Diaminobenzidine
DMH	Dorsomedial hypothalamic nucleus
GluRs	Glutamate receptor subunits
LH	Lateral hypothalamic area
NMDA	N-methyl-D-aspartate
PFA	Paraformaldehyde
PVN	Paraventricular nucleus
SON	Supraoptic nucleus
VMH	Ventromedial hypothalamic nucleus
